# *Eeyore*: A Novel Mouse Model of Hereditary Deafness

**DOI:** 10.1371/journal.pone.0074243

**Published:** 2013-09-23

**Authors:** Kerry A. Miller, Louise H. Williams, Hans-Henrik M. Dahl, Shehnaaz S. M. Manji

**Affiliations:** 1 Genetic Hearing Research, Murdoch Childrens Research Institute, Melbourne, Victoria, Australia; 2 The HEARing CRC, Audiology, Hearing and Speech Sciences, University of Melbourne, Melbourne, Victoria, Australia; 3 Department of Paediatrics, University of Melbourne, Melbourne, Victoria, Australia; 4 Department of Otolaryngology, University of Melbourne, Royal Victorian Eye and Ear Hospital, East Melbourne, Victoria, Australia; University of Washington, Institute for Stem Cells and Regenerative Medicine, United States of America

## Abstract

Animal models that recapitulate human disease are proving to be an invaluable tool in the identification of novel disease-associated genes. These models can improve our understanding of the complex genetic mechanisms involved in disease and provide a basis to guide therapeutic strategies to combat these conditions. We have identified a novel mouse model of non-syndromic sensorineural hearing loss with linkage to a region on chromosome 18. *Eeyore* mutant mice have early onset progressive hearing impairment and show abnormal structure of the sensory epithelium from as early as 4 weeks of age. Ultrastructural and histological analyses show irregular hair cell structure and degeneration of the sensory hair bundles in the cochlea. The identification of new genes involved in hearing is central to understanding the complex genetic pathways involved in the hearing process and the loci at which these pathways are interrupted in people with a genetic hearing loss. We therefore discuss possible candidate genes within the linkage region identified in *eeyore* that may underlie the deafness phenotype in these mice. *Eeyore* provides a new model of hereditary sensorineural deafness and will be an important tool in the search for novel deafness genes.

## Introduction

Despite advances in our knowledge of genes that contribute to human disease, in many cases the causative gene remains unknown. The use of animal models in the search for causative disease genes has been revolutionary in providing insights into complex mammalian developmental and genetic pathways [1], [2]. Mouse models are often used to elucidate the role of genes in human hearing loss as investigations of the auditory system can be extrapolated across species due to the high conservation of structures such as the cochlea [3], [4]. An accurate and well-characterised mouse model is also necessary for the development of novel treatments and studies into the relationship played between genetics and the environment in conditions such as deafness with a multiplicity of aetiologies.

Hearing loss is the most prevalent sensory impairment in man, affecting as many as 1 in 500 newborns [5], with many suffering a progressive loss [6]. A fully functional auditory system in humans is necessary for communication and perception of the surrounding environment. Even a minor level of hearing loss can impact on lifelong social, financial, vocational and education needs [7]. Hearing loss aetiology is largely genetic, where 70% of non-syndromic congenital deafness is caused by a genetic mutation [5]. The extreme heterogeneity of this condition reflects the complexity of the human auditory system, with over 200 genes thought to contribute to audition [8]. To date, 70 genes have been shown to play a role in non-syndromic, hereditary hearing loss (NSHL), which can be transmitted by autosomal, recessive, dominant, sex-linked or mitochondrial modes of inheritance (http://hereditaryhearingloss.org/). In addition, the same gene can cause syndromic or non-syndromic forms of deafness and some recessive forms can even be caused by two mutations in different genes that are from the same functional group [9]. Inherited hearing loss is non-syndromic in 70% of reported cases, where 80% of these are inherited in an autosomal recessive pattern [5], [10]. In order to fully explore the fundamental molecular and biological pathways required to maintain normal hearing, the identification of new animal models that mimic the hearing loss seen in humans is imperative for research into this domain.

We have identified a novel mouse model of non-syndromic, sensorineural hearing loss via a comprehensive ENU (*N*-ethyl-*N*-nitrosourea) mouse screen at the Australian Phenomics Facility (APF). The hearing loss is congenital in these mice and affected progeny show abnormal structural morphology within the cochlear sensory regions. The causative mutation is inherited as a single recessive locus on chromosome 18, between 12,368,762 and 33,968,545 Mb. To date no genes in this region have been associated with human hearing loss. Genes within this linkage region are therefore novel candidates for human hereditary hearing loss and identification of the causative mutation will provide additional insights into the genetic pathways responsible for the correct maintenance of hearing.

## Methods

### The *eeyore* mouse

All procedures were approved by the Royal Children's Hospital Animal Ethics Committee, RCH AEEC #A585. Mice were screened for hearing loss as part of a large-scale ENU mutagenesis program at the APF, as described previously [11]. Frozen embryos and sperm from the *eeyore* strain are stored at the Transgenic Animal Service of Queensland (TASQ), Australia (http://tasq.uq.edu.au/).

### Hearing Tests

Clickbox observations and Auditory Brainstem Responses (ABR) were used to screen for hearing loss in *eeyore* mice, as previously described [11]. Data were analysed using a non-paired T-test and analysis of variance. Mice were also screened for behaviour associated with a vestibular dysfunction, by observation of circling and head tossing/star-gazing behaviours.

### Genetic Mapping and DNA Sequencing

Mutant *eeyore* mice were identified as having a severe hearing loss by the tests described above. Affected *eeyore* mice were outcrossed to the BALB/c mapping strain and bred to produce affected F2 offspring. Genomic DNA from tails of hearing and deaf littermates was isolated by Proteinase K digestion and phenol/chloroform extraction to use for homozygosity mapping. DNA from 20 affected *eeyore* mice were analysed using Sequenome MassARRAY (AGRF). The deafness locus was mapped using methods described previously [11]. Additional informative SNPs were identified using MGI [12] and affected *eeyore* mice were analysed using SNP-specific primers designed using Primer3 [13]. DNA was amplified with GoTaq® Flexi DNA Polymerase (Promega) using the following cycling conditions; 35 cycles of 95°C for 30 s, 58°C for 30 s and 72°C for 30 s. PCR products were sequenced with a BigDye^TM^ v3.1 Terminator Cycle Sequencing Kit (Applied Biosystems) and products read using an ABI 3130×l capillary genetic analyser (Applied Biosystems). Sequencing chromatograms were compared to the published gDNA sequence using Mutation Surveyor (v2.60) software and the genotype of affected mice determined. Using the UCSC genome browser [14] linkage intervals were examined to identify top candidate genes within the linkage region.

### Tissue Collection

Mice were anaesthetised with isoflurane and culled by cervical dislocation according to the National Health and Medical Research Council Australian code of practice for the care and use of animals for scientific purposes (RCH AEEC approval #A585). Adult mouse cochleae were dissected and processed as described [15]–[17].

Ossicles were dissected from half heads of adult (14 week old) hearing and deaf *eeyore* littermates. Briefly, the middle ear was exposed by dissection of the bulla and removal of the tympanic membrane, taking care not to damage the malleus underneath. The malleus, incus and stapes were removed from the middle ear with care, stored in PBS and photographed with a Leica DC200 camera (Leica Microsystems Ltd).

### Histology & Scanning Electron Microscopy (SEM)

Cochleae were isolated from 8, 12 and 24 week hearing and deaf *eeyore* mice and processed for H&E staining as described previously [18]. A standard H&E protocol was followed with a 4–5 min incubation in hematoxylin and 45 s staining in eosin then mounted with Entellan® (Merck). Images were taken on a Nikon Eclipse 80i microscope (Pathtech).

Cochleae from 12–14 week old mice were dissected, fixed and processed for SEM as previously described [15]. Tissues were viewed using a Philips XL30 FE scanning electron microscope.

## Results

### Affected *eeyore* mice have a severe progressive hearing loss

**Figure 1:**
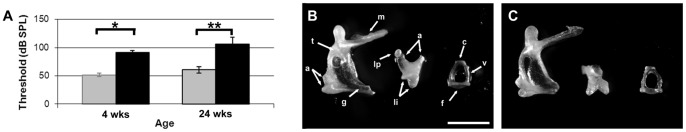
Hearing Profile (A) and middle ear bone dissections (B–C) of *eeyore* mice. ABR thresholds of hearing (grey bars) and deaf (black bars) *eeyore* littermates at 4 (*p = 4.8×10^−10^) and 24 (**p = 1.2×10^−10^) weeks of age. The ossicles appear largely normal in deaf mice (C), comparable to normal morphology of the malleus, incus and stapes in normal hearing mice (B). M; manubrium of malleus, A; articulation surfaces of malleus and incus joint, T; tubercle, G; gonial angle, LI; attachment points of suspensory ligaments of incus, LP; lenticular process, C; capitulum of stapes, V; arched ventral crus, F; footplate. Scale bar: 1 mm.

Auditory function in *eeyore* mice was measured by ABR thresholds. Affected mice showed a severe hearing loss at 4 weeks of age (85–95 dB SPL; Figure 1A). By 12 weeks of age the hearing loss has deteriorated and affected mice are profoundly deaf by 24 weeks (100–120 dB SPL). Hearing littermates show normal ABR thresholds at 4 weeks, but a slight increase was recorded by 24 weeks, which may be attributed to inherent age-related hearing loss (AHL) in the C57BL/6J strain. No circling behaviour indicative of a vestibular dysfunction was identified in deaf mice. Affected mice showed no signs of health deterioration, suggesting no other associated abnormalities in this strain. Affected founders lived to 20 months of age and all other affected mice lived beyond 6 months without any health concerns.

### Middle ear morphology is normal in *eeyore* mice

Middle ear defects can also be associated with elevated ABR thresholds. To determine whether the hearing loss in *eeyore* was due to a disruption in conductance through the middle ear we examined the middle ear bones. No evidence of infection was seen in deaf mice and the tympanic membrane and bulla were normal. Detailed examination of the ossicles did not highlight any structural differences of the malleus, incus or stapes in deaf mice (Figure 1C) when compared to ossicles from normal hearing littermates (Figure 1B). Normal ossicle formation indicates a sensorineural hearing loss in *eeyore*.

### Histopathology of *eeyore* mice

**Figure 2:**
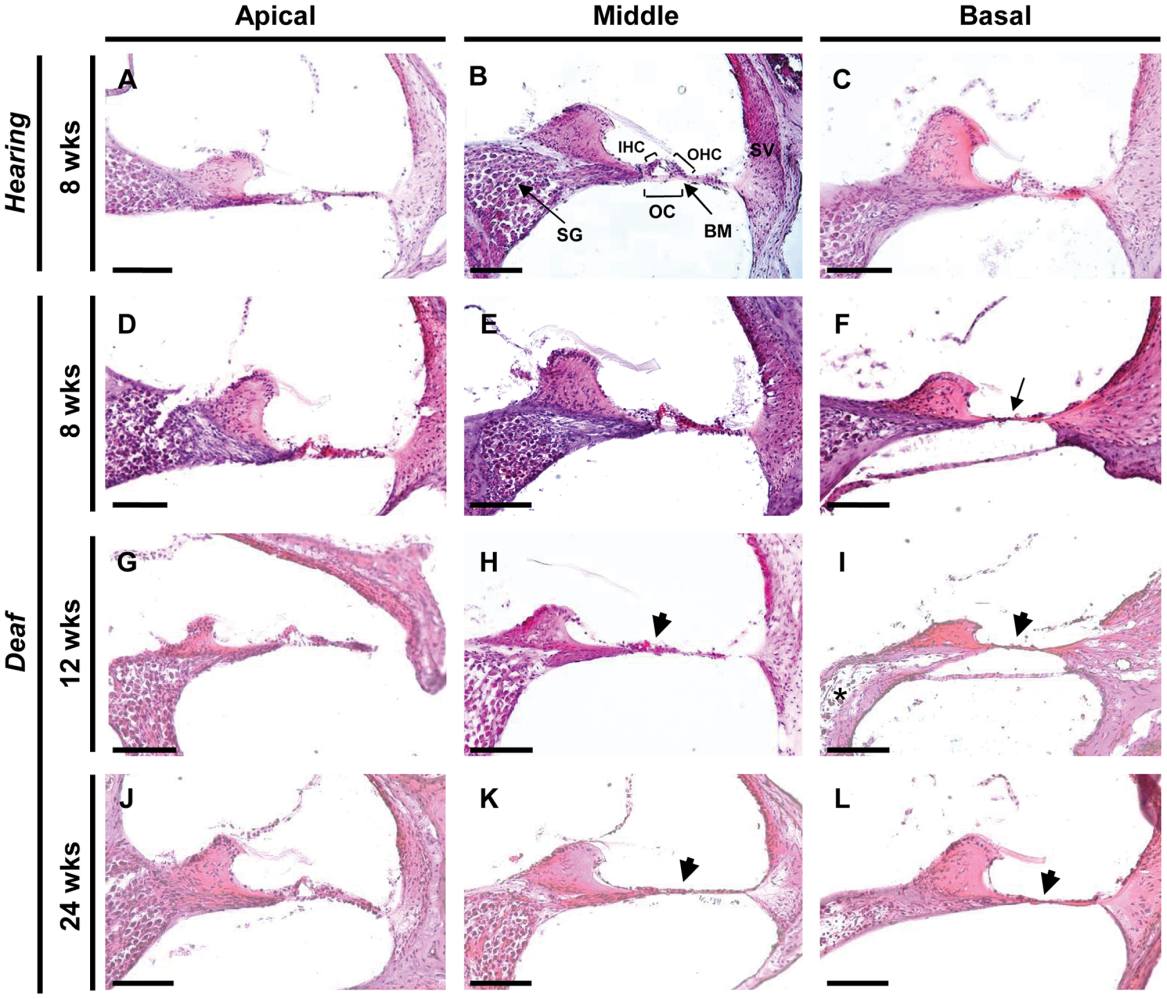
Haematoxylin and Eosin (H&E) staining of cochlea sections from 8 week old hearing (A–C) and 8–24 week deaf (D–L) mice at the apical, middle and basal levels. Normal cochlear morphology shows an intact organ of Corti and the presence of a tunnel containing inner and outer hair cells and intact spiral ganglion and stria vascularis (B). Collapse of the tunnel of Corti is evident by 8 weeks at the basal level in deaf mice (arrow in F), and degeneration of the spiral ganglion is apparent at the basal level by 12 weeks of age (asterix in I). SG, spiral ganglion; OHC, outer hair cells; IHC, inner hair cells; OC, organ of Corti; BM, basilar membrane. Scale bar: 100 μM.

Gross examination of cochleae from deaf *eeyore* mice did not highlight any visible structural changes. Closer examination of the inner ear by H&E staining revealed the structural integrity of the cochlea is compromised in deaf mice when compared to hearing controls (Figure 2). Degeneration of the inner ear is evidently progressive as the tunnel of Corti (toC) is missing in the basal level of the cochlea at 8 weeks (Figure 2F), that extends to the middle regions of deaf mice with age (arrowhead in Figure 2H, I, K and L). Although the spiral ganglion appears to show some degeneration in places (Figure 2I), the stria vascularis appears normal at all levels of the cochlea in deaf mice. Most evident in the basal cochlear region, degeneration of spiral ganglion neurons is likely to be a secondary consequence to the collapse of the organ of Corti and loss of sensory cells within the cochlea.

### Abnormal stereocilia morphology in deaf *eeyore* mice

**Figure 3:**
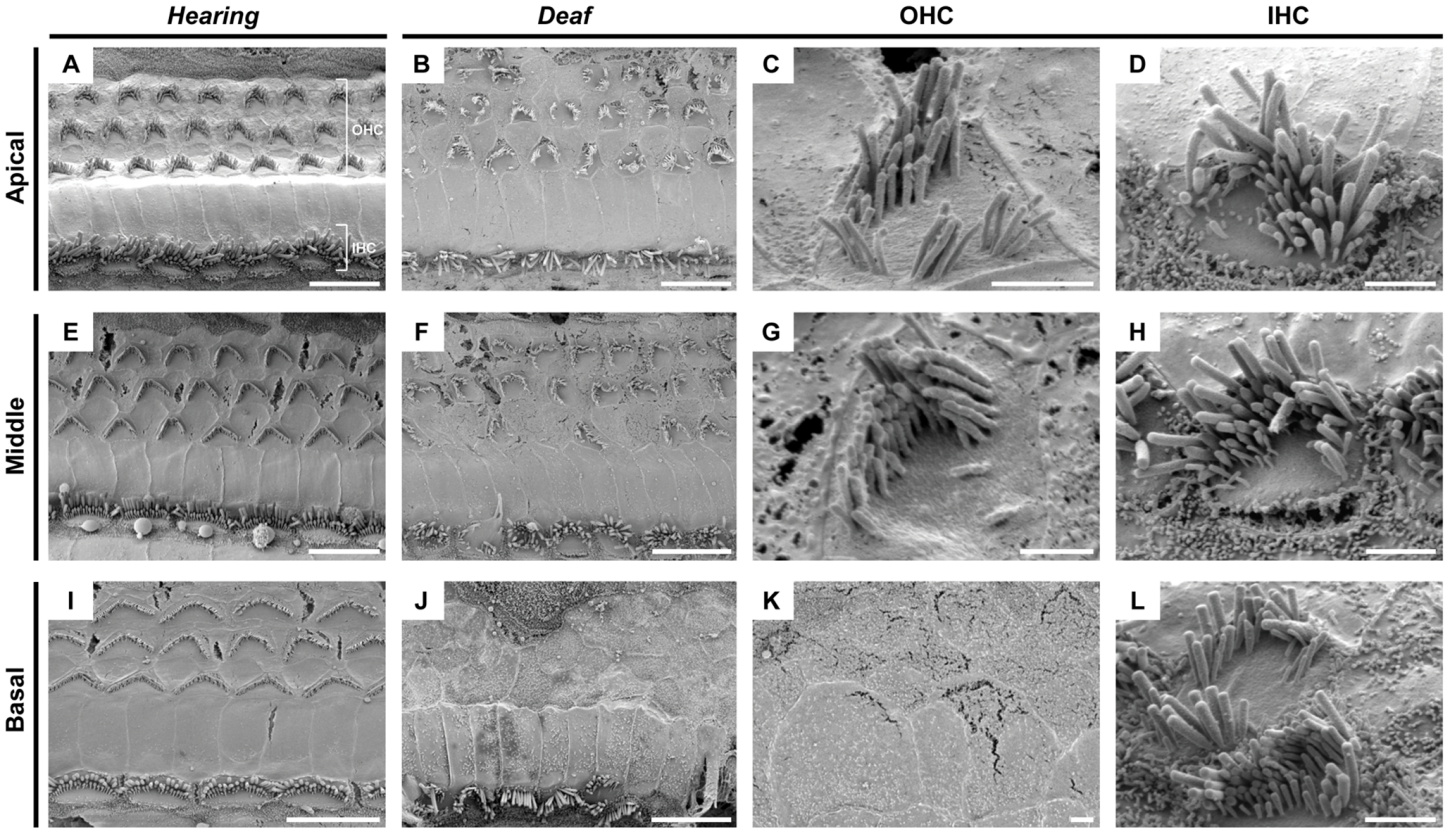
Scanning electron micrographs of cochlear sensory epithelium from 12–14 week old hearing (A, E and I) and deaf (B–D, F–H, J–L) mice at the apical, middle and basal cochlear level. OHC bundles are disorganised and misorientated at the middle and basal levels of the cochlea. Bundles are completely missing at the basal level at this age. IHC have an abnormal morphology, appearing ‘OHC-like’ in structure. OHC, outer hair cells; IHC, inner hair cells. Scale bar: 10 μM (A, B, E, F, I and J), 2 μM (C, D, G, H, K and L).

Ultrastructural analysis of cochlear sensory organs from hearing and deaf mice by SEM identified morphological changes in hair cell stereocilia in deaf mice when compared to hearing mice (Figure 3). At 14 weeks of age, degeneration of outer hair cells (OHC) is observed at all levels of the cochlea, most evident at the basal turn where no OHC stereocilia are present (Figure 3J). OHC bundles at the apical and middle levels do show signs of degeneration as they have an abnormal structure, often mis-orientated and disorganised (Figure 3B and F). These bundles appear non-uniform in height and are frequently missing stereocilia. Inner hair cells (IHC) show an abnormal morphology in that they look almost OHC-like in their appearance (Figure 3D, H and L). WT IHC bundles are aligned in a relatively straight orientation, whereas affected IHC bundles look ‘U-shaped’, often with splayed stereocilia. Some IHC also appear to have two rows of stereocilia bundles per cell (Figure 3L).

### SNP analysis reveals a novel deafness locus in *eeyore*

**Figure 4:**
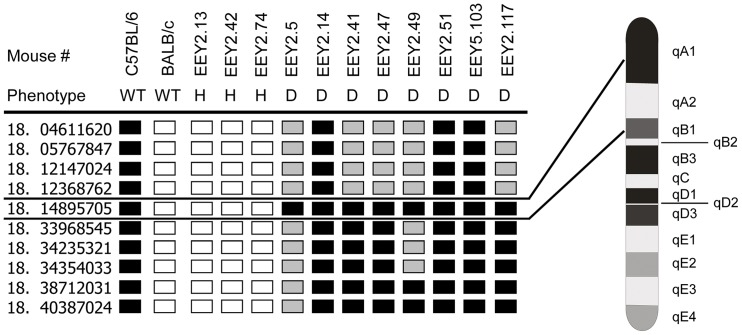
SNP mapping of the mutation in *eeyore* mice. DNA from 3 hearing (H) and 8 of 16 deaf (D) mice were analysed for SNP markers on chromosome 18, which are listed in the first column and indicate megabase position (4.6–40.3 Mb). The mouse identification numbers and phenotypes are indicated at the top of each column. The genotype of each mouse is either homozygous for C57BL/6 (black) or BALB/c (white) or heterozygous (grey) for that marker. On the right is a diagram of mouse chromosome 18 showing the interval to which the deafness phenotype mapped to.

The heritability of the deafness phenotype in F2 progeny was consistent with a recessive inheritance trait, with approximately 25% of offspring affected (14/56). SNP analysis in the *eeyore* strain mapped the minimal causative deafness region to a locus on mouse chromosome 18 between markers rs8255001 and rs3696933 (Figure 4), based on NCBI build 37. The critical region was analysed for known genes, ESTs, predicted genes and hypothetical proteins by using the NCBI Genome Browser (URL: http://www.ncbi.nlm.nih.gov), the Ensembl Genome Browser (URL: http://www.ensembl.org), and the UCSC Genome Browser (URL: http://www.genome.ucsc.edu). This 21.6 Mb region contains 143 genes that were analysed for likelihood of causing the deafness phenotype using the program PosMed [19], and prioritised against genes known to be responsible for NSHL. PosMed utilises the GRASE database search engine to identify and rank candidate genes [20]. The linkage region shares synteny with human chromosome regions 2:127522104–128415264, 5:110433680–112101480, 18:19337472–20895888 and 18:21850216–39101856 and no previous mutations within these regions have been identified in human non-syndromic hearing loss.

## Discussion and Conclusions

The causative locus identified in *eeyore* spans 21.6 Mb of chromosome 18 and contains 143 genes, each of which may contribute to the phenotype identified in our mouse model. Candidate genes ranked by PosMed were examined to identify their known function and likelihood of causing the deafness phenotype in *eeyore* mice. The top-ranked candidate genes within this region are listed in Table 1.

**Table 1:** Top-ranked candidate genes in the *eeyore* genetic interval.

*Gene Symbol*	*NCBI Accession number*	*Exons*	*Strand*	*Location*	*Gene name*	*Protein Function*	*Human Disease*	*Reference*
***Aqp4***	NM_001650.4	5	Rev	18: 15,547,903–15,562,193	Aquaporin 4	Mercurial-insensitive water channel	Neuromyelitis optica	[45], [46]
***Npc1***	NM_008720.2	25	Rev	18: 12,348,202–12,394,909	Niemann Pick type C1	Vesicular trafficking at nerve terminals	Niemann-Pick disease type C1	[47], [48]
***Ttr***	NM_013697.5	4	For	18: 20,823,751–20,832,825	Transthyretin	Thyroxine plasma transport protien	Amyloidosis	[49], [50]
***Ercc3***	NM_133658.1	15	For	18: 32,399,954–32,429,805	Excision repair cross-complementing rodent repair deficiency, complementation group 3	DNA repair, Transcription	Xeroderma pigmentosum group B	[51], [52]
***Tslp***	NM_021367.2	5	For	18: 32,975,037–32,979,453	Thymic stromal lymphoprotein	Inflammation response	Eosinophilic esophagitis	[53], [54]
***Dsg3***	NM_030596.3	16	For	18: 20,668,805–20,699,811	Desmoglein 3	Cell-cell adhesion	Pemphigus Vulgaris	[55]
***Cdh2***	NM_007664.4	16	Rev	18: 16,747,386–16,967,755	Cadherin 2, N-cadherin	Calcium-ion-dependent adhesion	Neuroblastoma	[56], [57]
***Hrh4***	NM_153087.2	3	For	18: 13,165,499–13,181,391	Hystamine H4 receptor	G-protein coupled receptor	None known	[58], [59]
***Pik3c3***	NM_181414.5	25	For	18: 30,432,401–30,507,780	Phosphoinositide-3-kinase, class 3	Receptor-mediated signal transduction, intracellular trafficking	None known	[60]
***Lama3***	NM_010680.1	75	For	18: 12,492,533–12,741,522	Laminin, alpha 3	Cell adhesion	Junctional epidermolysis bullosa, Laryngoonychocutaneous syndrome	[61], [62]
***Myo7b***	NM_032394.3	47	Rev	18: 32,118,888–32,196,615	Myosin VIIb	Molecular motor	None known	[63], [64]

Of particular interested are candidate genes that have previously been associated with deafness such the water-selective membrane channel Aquaporin 4 (Aqp4). Expression of this gene has been reported within the cochlear sensory cell epithelium and glial cells of the auditory nerve [21], [22], an elevated auditory brainstem threshold was recorded in an Aqp4-null mouse model [21] and a human variation in Aqp4 was recently linked to hearing loss in a cohort of patients with non-syndromic hearing loss [23].

Cadherin 2 (Cdh2 or N-cadherin), the class III phosphoinositide 3-kinase (PIK3C3) and Myosin 7b (Myo7b) are also plausible candidates within this linkage region as they all have family members that have been identified in deaf patients or mouse models of hearing loss. The cadherin family member, Cadherin 23 (Cdh23), is a major component of sensory hair cell tip links [24]–[26] and mutations in Cdh23 are responsible for the human deafness type DFNB12 [27] and Usher Syndrome Type 1D [27]–[29]. A number of analogous mouse models to these conditions have been reported [15], [30]–[35]. Several members of the phosphoinositide synthesis pathway have been reported in the literature to play an important role in hearing [36]–[39] and we recently identified a mouse model of progressive sensorineural hearing loss with a mutation in another member of this pathway, Synaptojanin 2 (Synj2) [11]. The myosin family member Myosin 7a (Myo7a) is well known to underlie the human deafness conditions DFNA11, DFNB2 and USH1B [40] and a number of mouse models of deafness with mutations in Myo7a have been reported [33], [41]–[44].

Our *eeyore* mice represent a novel model for studying progressive sensorineural recessively inherited hearing loss, common in the human population but with limited information on its molecular basis. *Eeyore* mutants are severely deaf at an early age which progresses to profound deafness within a number of weeks. Morphological and structural defects are detected within the cochlea indicating sensorineural deafness in these mice. The critical region localizes to a novel 21.6 Mb region on chromosome 18, containing a number of genes that could be implicated in the pathogenicity of this disorder. The underlying genetic mutation and mechanism remains to be elucidated, however this region has not previously been associated with recessively inherited non-syndromic deafness in humans. The rapid speed and reduced costs of targeted genome sequence analyses in mouse models of human disease, could potentially lead to the identification of the molecular mutation responsible for the hearing impairment in *eeyore*. The identification and subsequent characterisation of mouse models such as *eeyore* is fundamental to our understanding of the molecular mechanisms involved in normal hearing, and where this goes wrong in hearing impairment. We therefore report *eeyore* as a novel mouse model of NSHL that will provide new insight into the genetic mechanism underlying progressive and age-related hearing loss, and contribute to the validation and development of novel therapeutics when corresponding genetic lesions are identified within the human genome.
